# Editorial: Post-traumatic Stress in the Family

**DOI:** 10.3389/fpsyg.2018.00040

**Published:** 2018-02-06

**Authors:** Danny Horesh, Adam D. Brown

**Affiliations:** ^1^Department of Psychology, Bar-Ilan University, Ramat Gan, Israel; ^2^Department of Psychiatry, New York University, New York City, NY, United States; ^3^Department of Psychology, Sarah Lawrence College, Bronxville, NY, United States

**Keywords:** traumatology, family, intergenerational transmission, PTSD, parenthood, marriage

Traumatic events may have severe, long-lasting effects on the individual survivor. These include, first and foremost, post-traumatic stress disorder (PTSD)—a chronic, debilitating systemic psychiatric disorder, characterized by symptoms of re-experiencing, avoidance, negative mood and cognitions, and autonomic hyper-arousal (American Psychiatric Association, 2013)—and other comorbid conditions, including major depression, anxiety, substance abuse, and somatization (Galatzer-Levy et al., 2013).

During the past few decades, studies have shown that individuals in the trauma survivor's close proximity may also suffer from psychological symptoms associated with his/her experience. Extant research reveals trauma's massive influence on the entire family system, through processes related to parenthood, spousal relationships and household conflict and aggression (Galovski and Lyons, 2004). In addition, trauma was often found to be “contagious,” as manifested in numerous studies of secondary traumatization (e.g., Dekel et al., 2016), i.e., post-traumatic symptoms experienced by those who were only indirectly exposed to trauma, whether by hearing stories about the event, or by witnessing their negative effects on their loved one (Figley, 1995).

Nonetheless, major gaps in knowledge still exist regarding trauma's effects on families, couples, and parent-child dyads. This is perhaps most apparent when one considers the relative scarcity of treatment modules directly targeting the post-traumatic family (e.g., Berkowitz et al., 2011), compared to the large variety of evidence-based treatment options offered to the individual survivor (Kar, 2011). In addition, much remains to be understood regarding the underlying mechanisms of post-traumatic distress in the family, and the deep dynamics between family members following traumatic exposure.

This Research Topic aims to present readers with new, updated empirical, and theoretical perspectives about post-traumatic stress within the family system. The Topic gathers scholars from around the globe, working in diverse post-traumatic contexts (military and civilian) and age groups (children, adults, and the elderly). The 10 papers published in this Topic may generally be divided into two categories: studies examining trauma's effects on couples, and others looking at the child-parent dyad. We will now present these papers, starting with those focusing on couples.

In her clear, concise, opinion piece, Vilchinsky presents a strong and important argument, calling for more family research in the field of cardiac-disease-induced PTSD (CDI-PTSD). More specifically, she notes that although cardiac diseases are highly prevalent, their post-traumatic effects on the patient's spouse have been widely neglected over the years. Studies suggest that 12–15% of all patients who undergo an acute coronary event (ACE) subsequently develop CDI-PTSD. Since one's partner is often the main caregiver, and given the fact that partners often directly or indirectly witness the incidence of ACEs or subsequent hospitalizations, they face an increased risk of suffering from post-traumatic symptoms and emotional distress in general. Thus, there is a need for much more research and clinical work in this field.

Two papers in the Topic attempt to disentangle complex questions related to couple dynamics in the face of trauma. In their paper, Misca and Forgey critically review the literature on bidirectional intimate partner violence (IPV) among military and veteran populations, and its association with PTSD. Perhaps unsurprisingly, studies reveal a vicious cycle of mutual aggression between soldiers/veterans and their spouses. However, two main critiques emerge from the review. First, none of the studies reviewed have explored the partner's PTSD and its possible role in the bi-directional nature of violence. Second, the authors highlight the importance of developing a comprehensive measure of IPV, tapping its frequency, physical and emotional impact, and motives. Future studies that would follow these recommendations may enhance our understanding of IPV following trauma. In the second paper, Lahav et al. present novel, even provocative, findings regarding post-traumatic growth (PTG) among ex-POWs and their spouses. They employ a longitudinal study design to show that PTG, traditionally viewed as a positive, salutogenic phenomenon, may in fact predict adverse dyadic outcomes. The authors infer that PTG reflects defensive, illusory beliefs, rather than an authentic process of personal development following trauma.

A study by Hershkowitz et al. serves as a possible bridge between the two categories of papers included in this Topic, as it sheds light on processes related to both parenthood and spousal relationships. Its results show that PTSD is negatively associated with parenting behaviors. More importantly, results show that marital satisfaction may buffer the effects of depression on parenting, thus connecting between two areas of family life.

As noted, several papers included in this Topic focused on the parent-child dyad. Two of these papers examined parents' potential role as “stress regulators” for their children. Wise and Delahanty's review paper clearly reveals that a parent's post-traumatic symptoms are associated with his/her child's distress following traumatic injury. The review also underscores the importance of discussing, in an emotionally open and supportive manner, the traumatic event within the family. Also, given the impact of moderators such as parent/child gender, and the developmental stage of the child, it is highly unlikely that a singular, one-size-fits-all approach to treatment interventions for parents of injured children will be effective in reducing child symptoms. In another paper, Slone and Shoshani report an interesting gender effect, where the mother's parenting style—but not the father's—moderated the association between exposure to political violence and emotional distress among Israeli adolescents. More specifically, maternal authoritative parenting style and warmth emerged as potent protectors against the child's mental health symptoms.

Three studies included in this Topic focus on various aspects of inter-generational transmission of trauma, and the general effect of parental PTSD on the parent-child relationship. Creech and Misca's review paper reveals the vast influence of military-related parental PTSD on the quality of the parent-child bond and on children's well-being, with an emphasis on internalizing-externalizing behaviors. Once again, parental gender issues are discussed, and those seem to merit more research in the future. A study by Shrira et al. focused on a unique sample—aging individuals who are second generation to Holocaust survivors. The study's important findings reveal the far-reaching implications of parents' trauma, as manifested in their offspring's negative aging processes, more than half a century after the Holocaust. Importantly, these effects seem to be associated with the offspring's secondary post-traumatic symptoms. In a highly original study, Kazlauskas et al. explore the concept of “inter-generational transmission of resilience,” as may be seen in sense of coherence among offspring of individuals suffering from post-traumatic symptoms. The idea that salutogenic processes may also be transmitted from one generation to another carries important clinical implications, and we wish to encourage future studies to further explore this area.

Finally, one study in this Topic examined another form of directionality, one stemming from the child and influencing the parent. Bitton et al. present findings attesting to parents' distress during their child's participation in ongoing military conflict. The study raises important questions regarding parents' peri-traumatic distress, as well as regarding the overlap between concepts such as parental anxiety, worry, and secondary post-traumatic symptoms.

To conclude, we wish to highlight two issues we view as highly important. The first has to do with the “geography” of trauma, and cross-cultural psychology in general. As can be seen in this Topic, and as explicitly noted by several of its authors, a large portion of the evidence regarding post-traumatic stress in the family comes from English speaking countries, most notably the U.S., as well as from Israel, a country continuously troubled by war and terror. We strongly encourage studies from other areas of the world, such as the one from Kazlauskas et al. (Lithuania), noted above. Finally, families are complex systems (Hill, 1949; McCubbin and Patterson, 1983), which, when facing stress or crisis, undergo dramatic changes involving parents, children, spouses, brothers, and their various inter-relations. This field therefore requires studies that would examine the family as a whole, in an attempt to shed light not only on the couple or parent-child post-traumatic dyad, but on the familial *gestalt*. Such studies would strengthen the existing impressive body of knowledge, and improve family-based interventions following traumatic events.

## Author contributions

DH and AB have equally contributed to all relevant tasks related to this paper. These include editing the research topic and preparing the editorial.

### Conflict of interest statement

The authors declare that the research was conducted in the absence of any commercial or financial relationships that could be construed as a potential conflict of interest.
